# Beyond the Classroom: The Influence of Food Insecurity, Mental Health, and Sleep Quality on University Students’ Academic Performance

**DOI:** 10.3390/foods13162508

**Published:** 2024-08-11

**Authors:** Alejandra Betancourt-Núñez, Rosa Díaz, Pablo Alejandro Nava-Amante, María Fernanda Bernal-Orozco, Andrés Díaz-López, Aaron González Palacios, Fabiola Márquez-Sandoval, Davis Velarde-Camaqui, Barbara Vizmanos

**Affiliations:** 1 Translational Nutrition Sciences, Department of Human Reproduction, Growth and Child Development Clinics, Centro Universitario de Ciencias de la Salud (CUCS), Universidad de Guadalajara (UdeG), Guadalajara 44340, Mexico; alejandra.bnunez@academicos.udg.mx (A.B.-N.); pablo.nava@alumnos.udg.mx (P.A.N.-A.); fernanda.bernal@academicos.udg.mx (M.F.B.-O.); yolanda.marquez@academicos.udg.mx (F.M.-S.); 2 Laboratory of Nutritional Status Evaluation, Department of Human Reproduction, Growth and Child Development Clinics, Centro Universitario de Ciencias de la Salud (CUCS), Universidad de Guadalajara (UdeG), Guadalajara 44340, Mexico; 3 Public Health Sciences, Department of Public Health, Centro Universitario de Ciencias de la Salud (CUCS), Universidad de Guadalajara (UdeG), Guadalajara 44340, Mexico; 4 Center for Educational Research and University Welfare, Department of Philosophical, Methodological and Instrumental Disciplines, Centro Universitario de Ciencias de la Salud (CUCS), Universidad de Guadalajara (UdeG), Guadalajara 44340, Mexico; aaron.gonzalez@academicos.udg.mx; 5 School of Humanities and Education, Tecnológico de Monterrey, Monterrey 64849, Mexico; a00832504@tec.mx (R.D.); davis.velarde@gmail.com (D.V.-C.); 6 Nutrition and Mental Health Research Group (NUTRISAM), Faculty of Medicine and Health Sciences, Universitat Rovira i Virgili (URV), 43201 Reus, Spain; andres.diaz@urv.cat; 7 School of Psychology, Universidad César Vallejo, Lima 22700, Peru

**Keywords:** food insecurity, academic performance, mental health, sleep quality, university students, depression, anxiety, stress

## Abstract

We aimed to analyze the association between food insecurity (FI) and academic performance in university students, including mental health and sleep quality (SQ), in an association model. A cross-sectional design included university students (*n* = 466, 72.5% women) from Mexico. We applied the Latin American and Caribbean Food Security Scale, the Depression Anxiety and Stress Scale (DASS-21), and the Pittsburgh Sleep Quality Index. Students self-reported their academic grading (AG) and perceptions about their academic performance. Spearman’s rho and multiple logistic regression models were used. Almost half (47%) experienced some level of FI. The median AG was 95. AG was not significantly (*p* > 0.05) correlated with FI, nor with depression, anxiety, stress, and SQ. Considering mental health and SQ, moderate/severe FI remained significantly associated with perceived overall progress in college (OR: 2.96; 95%CI: 1.49, 5.88) and attendance to classes (OR:3.14; 95%CI: 1.19, 8.28) as poor or regular, and it was positively related to perceiving difficulties in completing their studies (OR:2.75; 95%CI: 1.43, 5.29). Stress, anxiety, depression symptoms, and poor SQ were also significantly associated with poor/regular perception of academic performance. These findings highlight the need to address psychological and nutritional factors in university students to promote their well-being and academic success.

## 1. Introduction

Food insecurity (FI) is defined as the “inconsistent access to safe and nutritious food necessary for normal growth, development, and a healthy lifestyle, that arises from either a scarcity of food or inadequate resources to obtain it” [1]. FI severity varies widely. The latest report from The State of Food Security and Nutrition in the World indicates that approximately 29.6 percent of the global population experiences moderate to severe FI. Notably, Latin America and the Caribbean have been identified as regions with a significant burden of FI, where 37.5% of the population faces FI (24.9% moderate and 12.6% severe) [2]. Specifically in Mexico, according to Encuesta Nacional de Salud y Nutrición 2021 data (ENSANUT, Spanish acronym), 60.8% presented some level of FI, with 25.9% experiencing moderate or severe FI [3]. Among the general population, in different contexts, one of the groups severely affected by the presence of FI is university students [4,5,6]. In Mexico, the prevalence of any level of FI in university students was found to be 30.8% [6]. This prevalence is lower than the national, but nevertheless high. To our knowledge, only one study on the prevalence of FI in this population group in Mexico has been carried out by our research group [6].

The academic literature points to detrimental effects of FI on various outcomes in this segment of the population, like reported poorer health [5,7,8,9], lower intake of fruits and vegetables [5], less adherence to a dietary pattern rich in fruits, vegetables, and food rich in animal protein [10], skipping breakfast [5,11], less likeliness to have a healthy diet off campus [11], having poor mental health like depression, anxiety, and stress [9,11,12,13,14], and poor sleep quality [12,13]. Furthermore, in different geographical settings, a negative association between FI and academic performance has been shown in college students from the United States of America (US) [7,8,12,15,16,17,18], Australia [19], Malaysia [20], and university students around Europe [21]. Some reviews also show a negative association between FI and academic performance in college/university students [5,22].

In this regard, some studies have analyzed whether mental health and sleep quality mediate the association between FI and academic performance. Although these studies have identified a direct association between FI and academic performance, they have also shown that mental health statuses, such as depression, anxiety, and stress, are significant mediators in this association [15,19,20,23]. Additionally, sleep quality has been postulated as another potential mediator in the association between FI and academic performance among college students [24].

To our knowledge, there is no published research (observational or longitudinal design) that analyzes the association between FI and academic performance in university students from Latin American countries, including Mexico, Colombia, Brazil, Argentina, and Ecuador, among others. The context of university students may vary in each country. For example, in México, most university students (66.7%) live with relatives [6], compared to other places in which the students live alone or with roommates [12,15,20]. Also, the grading system can be varied in each country. In the different studies, the most used indicator to measure academic performance in college students from the US is the Grade Point Average (GPA, using a scale from 1 to 4) [5,7,8,12,15,16,17,18,23]. In college students from Australia, they used weighted average marks (WAM, score that range from 0 to 100) [19]. In México, academic grading (AG) employs a scale from 0 to 10 or 0 to 100.

Additionally, in any context, limited studies include mental health and sleep quality in the association model between FI and academic performance [24]. Therefore, the main objective of this study is to analyze the association between FI and academic performance in Mexican university students, including mental health and sleep quality in the association model.

Showing the possible association between FI and academic performance, considering mental health and sleep quality, firstly, will contribute to the limited evidence that exists in this regard for university students, and will lead to future studies with designs with a higher level of evidence. In addition, the results of this study may show the need for universities to monitor and generate programs to address FI, mental health, and sleep quality in university students in order to improve their academic performance. Finally, in Mexican university students, this study may be a milestone to highlight the importance of studying and addressing FI, considering that there is no evidence on this issue in this context.

## 2. Materials and Methods

### 2.1. Study Design, Data Collection, and Ethical Approval

This is a cross-sectional study, part of a larger project titled “Risk factors of food insecurity and their consequences on lifestyle and cardiovascular health in workers and students enrolled at a university”. Data collection took place over 20 months, from April 2022 to November 2023.

The sampling was non-probabilistic. We arbitrarily selected four groups of undergraduate students per semester (two groups from the morning shift and two from the afternoon shift) from each program of study in the health area (nursing, medicine, nutrition, and so on). We also included postgraduate students from the health area and undergraduate students from programs other than the health area who were interested in the study and wanted to participate. In addition, we selected university workplaces where access was authorized. We went to these classrooms and workplaces to carry out the invitation to study in person. We visited 199 classrooms with 20–25 students each (approximately 3980–4975 students received the invitation). Those who wanted to participate voluntarily registered online for the research project. The participants who registered to participate in this study were contacted online, and we sent their participant codes and questionnaires.

The research protocol for the overarching project was approved by the Committees of Research, Ethics in Research, and Biosafety of the University Center of Health Sciences, University of Guadalajara (Approval Number: CI-02322). Participation required voluntary acceptance and signing of the informed consent form.

### 2.2. Participants

In the present study, we included undergraduate and postgraduate students, mainly from the health area, enrolled in a public university located in western Mexico (*n* = 466). Specifically, we included undergraduate students from nutrition, dentistry, nursing, medicine, psychology, and physical culture programs (*n* = 426), and fewer students (*n* = 9) from computer engineering, physics, logistics and transportation engineering, law, pharmaceutical chemist-biologist, and forensic science. Students of different master’s and doctoral health-related programs were also included (*n* = 31). Participants included both male and female students who were over 18 years of age. The exclusion criteria were women who were pregnant or breastfeeding, individuals taking corticosteroids, isotretinoin, antiretrovirals, or danazol, and individuals diagnosed with cancer or those who had received treatment for cancer in recent months (these criteria were established in the macro project). For this analysis, students who were studying in the first semester were removed from the AG analysis because they did not yet have AG as university students (*n* = 65; 4 graduate students and 61 undergraduate students).

### 2.3. Measures

All participants were sent a link to complete an online survey about their sociodemographic information, food security status, mental health, sleep quality, and academic performance. The link first included the informed consent. Participants had access to the questions if they confirmed consent. Sociodemographic characteristics such as age, sex, semester of study, and work situation were included.

The Latin American and Caribbean Food Security Scale (ELCSA, Spanish acronym) [25] was used to measure FI. This validated scale for the Mexican population includes questions regarding the household’s experience with food access three months prior to the interview. The ELCSA comprises 15 items to which students respond with a yes (1 point) or no answer (0 points). If there are children under 18 years of age in the home, the 15 items are answered. If there are no minors under 18 years of age in the home, only the first eight questions are answered. Based on the scoring, FI was classified into four categories; the cut-off points are different for households without or with children under 18 years of age: food security (0 points), mild FI (1–3 or 1–5 points, respectively), moderate FI (4–6 points or 6–10 points, respectively), and severe FI (7–8 or 11–15 points, respectively).

Mental health statuses such as anxiety, depression, and stress were assessed using the Depression Anxiety and Stress Scale—21 (DASS-21), validated in Spanish [26]. This assessment comprises 21 items, each with four possible responses: did not apply to me at all (0 points); applied to me to some degree or some of the time (1 point); applied to me to a considerable degree or a good part of the time (2 points); applied to me very much or most of the time (3 points). The scores obtained in the items that measure stress, anxiety, and depression are added separately. The minimum and maximum scores of each emotional state are 0 to 21, with higher scores indicating more pronounced symptoms of these emotional states. This scale measures symptoms of stress, anxiety, and depression in the month prior to the study. DASS-21 has been applied in other studies to measure mental health in university students [20,27,28], reporting acceptable psychometric properties. In the present study, DASS-21 showed a Cronbach’s α coefficient of 0.937 on the total scale, 0.856 on the stress dimension, 0.903 on the depression dimension, and 0.828 on the anxiety dimension, reflecting acceptable internal consistency.

The Pittsburgh Sleep Quality Index (PSQI) [29] of 18 items, validated in Spanish, was employed to determine sleep quality. This scale is made up of seven components: (1) subjective sleep quality, (2) sleep latency, (3) sleep duration, (4) usual sleep efficiency, (5) sleep disturbances, (6) use of sleeping medications, and (7) daytime dysfunction. The score obtained in all these seven components is added (minimum score of 0, maximum score of 21). The higher the score, the worse the quality of sleep. This information can also be categorized into good quality (≤5 points) or poor quality (>5 points). This scale measures the quality of sleep in the month prior to the study. PSQI has also been used in other studies to measure sleep quality in university students, reporting acceptable validity indicators [12,13,27,28].

Regarding academic performance, the students self-reported their AG. This was calculated by averaging the grades obtained in all the units taken during their study career. In Mexico, AG can range from 0 to 100. A higher score is interpreted as better academic performance. Additionally, it is relevant to note that a score equal to or greater than 60 is considered a passing grade. Furthermore, the survey contained five questions about perceptions of academic performance [8], covering overall progress in college, attendance to classes, ability to pay attention in class, understanding of concepts, and perception of academic future. For each question, respondents could choose from four possible answers: “poor”, “regular”, “good”, or “excellent”. We grouped the poor and regular categories because the poor category was less frequent. Concerning the perception of academic futures, the response options are will successfully complete studies, will complete, but with difficulties, and will not complete the degree.

At the end of the questionnaires, participants received the interpretation of their food security and sleep quality situation via email, with recommendations corresponding to the results obtained. In the case of participants with some level of FI, in addition to recommendations for accessing food, they were given contacts for food programs to which they could turn if necessary. In the case of identifying significant alterations in the quality of sleep, the participant was suggested to go to a health professional for attention. Concerning mental health, the information was sent, on a second occasion, by mail, also, after having analyzed it, with a catalog of mental health institutions that could be of help to those who had high values of stress, depression, or anxiety.

### 2.4. Data Analysis

Descriptive analysis was performed with continuous variables expressed as median and 25th and 75th percentiles, while qualitative variables were presented as frequencies and percentages.

Chi-square tests were used to analyze differences in sociodemographic characteristics, FI, and perception of academic performance between men and women. Additionally, Chi-square tests were employed to estimate associations between the five items related to perceptions of academic performance and the different levels of FI. The differences in AG, depression, anxiety, stress, and sleep quality scores according to sex and perception of academic performance were analyzed using the Mann–Whitney U test and Kruskal–Wallis test, respectively, after verifying that these variables did not show a normal distribution.

We used Spearman’s rho correlations to examine the associations between FI, mental health, sleep quality, AG, and perception of academic performance. Furthermore, multiple logistic regression analyses were performed to evaluate the associations between FI and perception of academic performance, including mental health and sleep quality in the model, and adjusting for age, sex, and employment status in the total sample and excluding those in the first semester (because they had missing values of AG). All analyses were conducted using SPSS software, version 26, with a significance level set at *p* < 0.05.

## 3. Results

A total of 466 university students were included in this study. The median students’ age was 21 years; 72.5% were women, and 13.9% were from the first semester. Regarding occupation, 93.3% were undergraduate students, 6.7% were postgraduate students, and 33.9% combined studies with work. Around half of the students (47%) experienced some level of FI, and 79.8% reported poor sleep quality. Regarding academic performance, the median AG was 95. The perception of overall progress in university was mainly rated as good (55.4%) and excellent (30.5%). Class attendance was reported as excellent by 51.7% and good by 41.8%. The majority of students rated their ability to pay attention as good (59.4%), their understanding of the concepts taught in class as good (61.8%) and believed they would successfully complete their studies (85.2%). We identified a greater stress score in women, and they more frequently perceived their understanding of the concepts taught in class as poor/regular compared to men. Likewise, women had higher AG than men; however, this difference was not significant (*p* = 0.056) (Table 1).

A significant difference was observed in the perception (frequency of excellent, good, or poor/regular) of all indicators of academic performance, except for understanding concepts taught in class, according to the presence of food security/insecurity. No significant differences were observed in AG between the presence of food security/insecurity (*p* = 0.058); however, there was a tendency for AG to be higher in those who were food-secure (Table 2). We stratified these results by sex, considering differences in some academic performance variables between men and women. Particularly in women, the difference in perception of academic performance indicators according to the level of FI is maintained, while in men, some of these differences lose significance (Table S1).

Likewise, a significant difference was observed in the depression, anxiety, stress and quality of sleep scores between those who responded excellent, good, regular/poor in the academic performance indicators: overall progress in college, attendance to classes, ability to pay attention in class, and understanding concepts taught in class (Table 3).

Correlation analysis showed that AG was not significantly correlated with FI, depression, anxiety, stress, and sleep quality scores (Table 4). We stratified these correlation results by sex, considering the trend in the difference in AG and other academic performance variables between men and women. The correlations remained non-significant in men (Table S2). However, in women, AG was negatively correlated with sleep quality score (r = −0.128, *p* = 0.032); the higher the sleep quality score (that means poorer sleep quality), the lower the AG (Table S3).

Regarding other indicators of academic performance, perceptions about overall progress in college, attendance to classes, ability to pay attention in class, understanding concepts taught in class, and perception about academic future were negatively and significantly correlated with FI, depression, anxiety, stress, and sleep quality scores. This association means the lower the presence of symptoms of depression, anxiety, and stress, the lower the presence of FI, and the higher the sleep quality score (the lower the score, the higher sleep quality), the more positive perception in these academic performance indicators (Table 4). Furthermore, all these academic performance indicators, except the perception of the ability to pay attention in class, were positively and significantly correlated with AG (Table 4). When stratifying these results by sex, the correlations remained significant in women (Table S3), and many of these correlations lost significance in men (Table S2).

Further, FI was positively linked to depression, anxiety, stress, and sleep quality (Table 4). These correlations remained significant in women (Table S3), but not in men (Table S2).

Sleep quality was positively and significantly correlated with depression, anxiety, and stress (Table 4). This correlation remained significant in men and women (Tables S2 and S3).

In the multivariate model, in which FI, mental health, and sleep quality were included, we observed that mild FI (OR: 2.64; CI 95%: 1.32, 5.29), moderate/severe FI (OR: 2.96; CI 95%: 1.49, 5.88), and depression symptoms (OR: 1.10; CI 95%: 1.04, 1.16) were positively associated with poor/regular perception of overall progress in college, including graduating on time. Moderate/severe FI (OR: 3.14; CI 95%: 1.19, 8.28) and depression symptoms (OR: 1.10; CI 95%: 1.01, 1.19) were positively associated with poor/regular perception of attendance to classes. Moderate/severe FI (OR: 2.75; CI 95%: 1.43, 5.29), depression (OR: 1.09; CI 95%: 1.03, 1.16), anxiety (OR: 1.08; CI 95%: 1.01, 1.15), and stress symptoms (OR: 1.07; CI 95%: 1.002, 1.14) were positively associated with perceiving difficulties in completing their studies. In this model, FI was not significantly associated with poor/regular perception of understanding the concepts in class, nor with the ability to pay attention in class. Nevertheless, depression and anxiety symptoms were associated with poor/regular perception of understanding the concepts in class (OR: 1.08; CI 95%: 1.03, 1.13 and OR: 1.08; CI 95%: 1.02, 1.14, respectively). Depression symptoms and sleep quality were positively associated with poor/regular perception of the ability to pay attention in class (OR: 1.08; CI 95%: 1.03, 1.13 and OR: 1.14; CI 95%: 1.04, 1.24, respectively) (Table 5). These associations remained significant when first-semester students were excluded from the analysis (Table S4).

## 4. Discussion

To our knowledge, this is the first study that analyzes the association between FI and academic performance in university students from Latin American contexts, particularly in Mexico. Considering mental health and sleep quality in the analysis, we observed that moderate/severe FI remained positively and significantly associated with perceived overall progress in college and attendance to classes as poor or regular, and it was positively related to perceiving difficulties in completing their studies. FI was not significantly associated with poor/regular perception of the ability to pay attention in class and understand concepts in class. However, anxiety symptoms were associated with poor/regular perception of understanding concepts in class, and sleep quality was positively associated with poor/regular perception of the ability to pay attention in class. Depression symptoms were positively associated with perceived poor or regular overall progress in college, attendance to classes, understanding concepts in class, and the ability to pay attention in class. Stress, anxiety and depression symptoms were positively associated with perceiving difficulties in completing their studies. AG was not significantly correlated with FI, nor with depression, anxiety, stress, and sleep quality.

Presenting moderate or severe FI increases the probability that the university students perceive as poor or regular their overall progress in college (including the likelihood of graduating on time) and their attendance to classes. In addition, moderate or severe FI was positively related to perceiving difficulties in completing their studies. These results are consistent with qualitative studies on university students from the US, who reported that their food situation negatively affected their academic performance [30,31] and school attendance [31,32]. Also, some reviews have reported that the presence of FI negatively affects academic performance and that students with FI are more likely to miss classes or drop out of college [5,22]. This association may be due to the physical symptoms students suffer as a consequence of lack of access to food and irregular food consumption. In one study, students with marginal food security expressed difficulty in fully participating in their academics, having poor self-perception and a lack of motivation, as a consequence of, among other factors, their eating situation (skipped meals or eating unbalanced diets) and having physical symptoms such as fatigue, headaches, feeling less energized, sluggish or bloated, and hunger pains [30]. In another study, university students with some level of FI reported feeling tired and lacking energy due to hunger and that these physical manifestations of FI hinder good academic performance. Students also mentioned spending more time thinking about food than concentrating on their studies [31]. Also, university students with experiences of FI, hunger, or limited food resources frequently or intentionally refuse to go to class because their hunger situation makes them feel sick, have a lack of energy throughout the day, and feel dizzy [32]. In addition, some students have to work longer hours to earn more money, which compromises their study hours [31].

FI was not significantly associated with poor/regular perception of the ability to pay attention in class and understand the concepts in class. These results are different from reviews [5,22] and qualitative studies [30,32] in which it is reported that the presence of FI in university students negatively affects the comprehension of the contents seen in class and favors the inability to concentrate in class. However, depression symptoms and poor sleep quality were positively associated with poor/regular perception of the ability to pay attention in class. Moreover, depression and anxiety symptoms were positively associated with perceived poor/regular understanding of the concepts in class. In addition, depressive symptoms were also positively and significantly associated with perceived poor or regular overall progress at university and with perceived poor or regular class attendance. FI is positively associated with poor sleep quality [12,13] and poor mental health [9,11,12,13,14,15,19,20,23,32,33] in the present study, as well as in other studies conducted on university students. FI may promote sleep deprivation due to concerns about not having food access. In turn, a lack of quality sleep may lead to fatigue and, thus, an inability to concentrate or pay attention [31,34,35]. On the other hand, lack of access to food can be a major source of stress and anxiety, especially if this situation continues for an extended period of time [30,32]. Students may also experience stress or worry due to the impact FI has on their physical health and academic performance [32]. Symptoms of depression and anxiety can be a barrier to clear thinking [31]. It has also been shown that symptoms of anxiety, stress, and worry about their food and financial situation affect the academic performance of university students [31]. According to Maslow’s Hierarchy of Needs, basic physiological needs such as food and sleep must be met before individuals can focus on higher-order needs like learning and self-actualization. Our findings support this theory, as FI, poor sleep quality, and poor mental health, which disrupt these basic needs, were associated with negative perceptions of academic performance [36].

We observed that AG was not significantly correlated with FI, nor with depression, anxiety, stress, and sleep quality. This result contrasts with numerous studies from different geographical settings that have reported a significant negative association of FI [5,8,12,15,16,17,18,19,20,22] mental health [15,19,20,23] and sleep quality [24] with academic outcomes measured numerically in university students. A possible explanation for the lack of significant correlations in our study could be the reliability of self-reported academic performance, which may be influenced by biases such as social desirability, recall bias, and overestimation, since no evidence was requested. However, in other previous studies [8,12,15,20,23], the academic performance was self-reported, as in this study, obtaining significant results between FI and academic performance as measured by the score. The lack of association may also be due to the fact that the student was asked for his or her overall grade, i.e., taking into account grades from all semesters taken, and the instruments we used measure FI, mental health, and sleep quality in the short term (FI status in the last three months, mental health in the last week, and sleep quality in the last month). Two studies of US college students found a significant negative association between FI (12 months prior to the day of the survey) and cumulative GPA (average of all the GPAs achieved for each semester) [15,20]. In future studies, it would be better to obtain the score at the end of the semester during which the data were collected or to use instruments that assess long-term FI, mental health, and sleep quality. However, we consider the current study as an approach to the analysis of a problem that has not yet been studied in this context.

One of the limitations of this study is its cross-sectional design because causality cannot be determined. In future studies, a longitudinal study could be carried out to study the association between FI, stress, anxiety, and depression symptoms and poor sleep quality, occasional and sustained over time, with academic performance. Another limitation is that the participants were not randomly selected; therefore, the results cannot be generalized to the study universe. Furthermore, in our study, academic performance data are self-reported and may lend themselves to intentional or unintentional lying by the student about his or her AG or perception of academic performance. However, the participants were always reminded that their responses were confidential and that they would be disassociated from their personal data so they would feel comfortable answering truthfully. In future studies concerning grading, we suggest asking the student’s permission to request this information from the university. Finally, although men and women received the invitation to the survey equally, we had a greater participation of women than men.

One of the strengths of this study is that it adds to the limited evidence available in the Latin American context, particularly among Mexican university students. It leaves a precedent for future studies that could strengthen the evidence regarding the association of FI with academic performance. Another strength of this study is that it considered both objective and subjective measures of academic performance complementary. Most studies only include the association of FI with obtained grades as an indicator of academic performance [7,12,15,17,18,19,20]. Nevertheless, future research should use a mixed method (qualitative and quantitative) to understand this object of study entirely.

The practical implications of our findings suggest that universities should implement comprehensive support systems that address students’ psychological and nutritional needs to enhance students’ overall well-being and academic performance. This could be done by providing mental health services and nutritional assistance for free or at a low cost within educational settings and interventions aimed at improving sleep quality. Universities should develop the necessary infrastructure for hosting workshops and cafeterias to support the nutritional needs of students. Universities may also implement individual financial counseling and deliver on-campus food pantries. Based on the research findings of this study, we recommend that the government implement policies to support university students by funding workshops on mental health and sleep hygiene, as well as providing subsidies for low-cost meals in university cafeterias. Students are encouraged to proactively seek available support services and advocate for implementing comprehensive mental health and nutritional programs within their institutions.

## 5. Conclusions

We analyzed the association between FI and academic performance in university students, considering sleep quality and mental health. AG was not significantly correlated with FI, nor with depression, anxiety, stress, and sleep quality. However, FI and depression symptoms were associated with perceived overall progress in college and attendance to classes as poor or regular. Anxiety and depression symptoms were associated with poor/regular perception of understanding concepts in class. Poor sleep quality and depression symptoms were associated with poor/regular perception of the ability to pay attention in class. FI, stress, anxiety, and depression symptoms were associated with perceiving difficulties in completing their studies. These findings highlight the necessity for universities to develop comprehensive support systems that address students’ psychological and nutritional needs to promote their well-being and academic success.

## Figures and Tables

**Table 1:** University students’ characteristics and comparisons between female and male students.

	Total *n* = 466	Women *n* = 338 (72.5%)	Men *n* = 128 (27.5%)	*p*-Value
Age ^a^	21 (19, 22)	21 (19, 22)	20 (19, 22)	0.541
Type of student ^b^				0.678
Undergraduate student	435 (93.3)	314 (92.9)	121 (94.5)	
Postgraduate student	31 (6.7)	24 (7.1)	7 (5.5)	
Semester ^b^				0.881
First	65 (13.9)	48 (14.2)	17 (13.3)	
≥Second	401 (86.1)	290 (85.8)	111 (86.7)	
Hours worked per week ^b^				0.311
Do not work	308 (66.1)	227 (67.2)	81 (63.3)	
<10 h	59 (12.7)	45 (13.3)	14 (10.9)	
>11 h	99 (21.2)	66 (19.5)	33 (25.8)	
Food insecurity level ^b^				0.782
Food security	247 (53.0)	178 (52.7)	69 (53.9)	
Mild food insecurity	112 (24.0)	84 (24.9)	28 (21.9)	
Moderate/severe food insecurity	107 (23.0)	76 (22.5)	31 (24.2)	
Sleep quality score ^a^	8.0 (6, 10)	8.0 (6, 10)	8.0 (5, 10)	0.238
Sleep quality classification ^b^				0.084
Poor quality	356 (79.8)	267 (81.9)	89 (74.2)	
Good quality	90 (20.2)	59 (18.1)	31 (25.8)	
Depression ^a,c^	4.0 (2, 8)	5.0 (2, 9)	4.0 (2, 6)	0.093
Anxiety ^a,c^	5.0 (2, 9)	5.0 (2, 9)	4.0 (2, 7.5)	0.168
Stress ^a,c^	8.0 (5, 12)	9.0 (5, 12)	7.0 (4, 10)	<0.001
Academic grading ^a,d^	95.0 (92.6, 97.0)	95.0 (93.0, 96.9)	94.0 (90.0, 97.0)	0.056
How would you rate your overall progress in college, including graduating on time? ^b^				0.881
Excellent	142 (30.5)	101 (29.9)	41 (32.0)	
Good	258 (55.4)	188 (55.6)	70 (54.7)	
Poor/regular ^e^	66 (14.2)	49 (14.5)	17 (13.3)	
How would you rate your attendance (in-person or online) to classes? ^b^				0.239
Excellent	241 (51.7)	182 (53.8)	59 (46.1)	
Good	195 (41.8)	137 (40.5)	58 (45.3)	
Poor/regular ^e^	30 (6.4)	19 (5.6)	11 (8.6)	
How would you rate your ability to pay attention in class? ^b^				0.083
Excellent	58 (12.4)	35 (10.4)	23 (18.0)	
Good	277 (59.4)	205 (60.7)	72 (56.3)	
Poor/regular ^e^	131 (28.1)	98 (29.0)	33 (25.8)	
How would you rate your understanding of the concepts taught in class? ^b^				0.033
Excellent	63 (13.5)	39 (11.5)	24 (18.8)	
Good	288 (61.8)	207 (61.2)	81 (63.3)	
Poor/regular ^e^	115 (24.7)	92 (27.2)	23 (18.0)	
Regarding your academic future, you consider that you: ^b^				0.140
Will successfully complete studies	397 (85.2)	293 (86.7)	104 (81.3)	
Will complete, but with difficulties/not complete the degree ^f^	69 (14.8)	45 (13.3)	24 (18.8)	

^a^ The quantitative variables are expressed as median (25th 75th percentile). The Mann–Whitney U test was used to evaluate differences between women and men. A *p* < 0.05 was considered significant. ^b^ The qualitative variables are expressed as frequency (%). As applied, Chi-square or Fisher’s exact test was performed to evaluate differences between women and men. A *p* < 0.05 was considered significant. ^c^ Depression, anxiety, and stress scores range from 0 to 21. ^d^ In the analysis of academic grading, first-semester participants were excluded. This analysis was carried out in 401 students. ^e^ Because less than 2% responded to the “poor” response option, the “poor” and “regular” options were combined. ^f^ Because 0.9% responded to the “not complete the degree” response option, the “will complete, but with difficulties” and “not complete the degree” options were combined.

**Table 2:** Academic performance according to level of food security/insecurity.

	Food Security	Mild Food Insecurity	Moderate/Severe Food Insecurity	*p*-Value
How would you rate your overall progress in college, including graduating on time? ^a^				0.002 *
Excellent	89 (36.0)	28 (25.0)	25 (23.4)	
Good	137 (55.5)	61 (54.5)	60 (56.1)	
Poor/regular	21 (8.5)	23 (20.5)	22 (20.6)	
How would you rate your attendance (in-person or online) to classes? ^a^				0.002 *
Excellent	146 (59.1)	53 (47.3)	42 (39.3)	
Good	92 (37.2)	51 (45.5)	52 (48.6)	
Poor/regular	9 (3.6)	8 (7.1)	13 (12.1)	
How would you rate your ability to pay attention in class? ^a^				0.011 *
Excellent	40 (16.2)	12 (10.7)	6 (5.6)	
Good	151 (61.1)	62 (55.4)	64 (59.8)	
Poor/regular	56 (22.7)	38 (33.9)	37 (34.6)	
How would you rate your understanding of the concepts taught in class? ^a^				0.155
Excellent	40 (16.2)	15 (13.4)	8 (7.5)	
Good	154 (62.3)	66 (58.9)	68 (63.6)	
Poor/regular	53 (21.5)	31 (27.7)	31 (29.0)	
Regarding your academic future, you consider that you: ^a^				<0.001 *
Will successfully complete studies	223 (90.3)	95 (84.8)	79 (73.8)	
Will complete, but with difficulties/not complete the degree	24 (9.7)	17 (15.2)	28 (26.2)	
Academic grading ^b^	95 (93, 97)	94 (92, 96.3)	94 (90, 96.4)	0.058

^a^ The qualitative variables are expressed as frequency (%). Chi-square was performed to evaluate differences between food security/insecurity categories. ^b^ The quantitative variable is expressed as median (25th 75th percentile). Kruskal–Wallis was used to evaluate differences between food security/insecurity categories. * *p* < 0.05 was considered significant.

**Table 3:** Depression, anxiety, stress, and sleep quality score according to academic performance.

	Depression ^a^	Anxiety ^a^	Stress ^a^	Sleep Quality ^a^
How would you rate your overall progress in college, including graduating on time?				
Excellent	3 (1, 7) ***	4 (1, 7) ***	6 (3, 11) ***	7 (5.5, 9) ***
Good	4 (2, 9)	5 (2, 9)	9 (6, 12)	8 (6, 10)
Poor/regular	8 (4, 14)	7 (4, 10)	10 (7, 13)	9 (7, 11)
How would you rate your attendance (in-person or online) to classes?				
Excellent	4 (1, 7) ***	4 (2, 8) *	8 (4, 11) **	7 (6, 9) ***
Good	5 (2, 9)	5 (2, 9)	8.5 (6, 12)	8.5 (6, 10)
Poor/regular	7 (4, 14)	7 (3, 10)	10 (7, 14)	9.5 (7, 11)
How would you rate your ability to pay attention in class?				
Excellent	3 (1, 6) ***	3 (1, 6) ***	6 (2, 10) ***	7 (5, 9) ***
Good	4 (2, 7)	5 (2, 8)	8 (4, 11)	8 (6, 9.5)
Poor/regular	7 (3, 12)	6 (3, 10)	10 (7, 12)	9 (7, 11)
How would you rate your understanding of the concepts taught in class?				
Excellent	2 (1, 5) ***	3 (1, 7) ***	6.5 (3, 10) ***	7 (5, 8) ***
Good	4 (2, 7)	4 (2, 8)	8 (5, 11)	8 (6, 10)
Poor/regular	7 (4, 12)	7 (4, 11)	10 (7, 12)	9 (7, 11)
Regarding your academic future, you consider that you:				
Will successfully complete studies	4 (2, 7) ***	4 (2, 8) ***	8 (4, 11) ***	8 (6, 10) **
Will complete, but with difficulties/not complete the degree	8 (4, 12)	8 (4, 10)	11 (7, 13)	9 (7, 11)

^a^ The quantitative variables are expressed as median (25th 75th percentile). The Kruskal–Wallis and Mann–Whitney U tests were used to evaluate differences between perceptions of academic performance. * Significant at *p* < 0.05. ** Significant at *p* < 0.01. *** Significant at *p* < 0.001.

**Table 4:** Correlations between academic performance and food insecurity, sleep quality, depression, anxiety, and stress symptoms in university students (*n* = 466).

	Food Insecurity	Academic Grading ^a^	Perception about Overall Progress in College	Perception about Attendance to Classes	Perception about the Ability to Pay Attention in Class	Perception about Understanding Concepts in Class	Perception about Academic Future	Depression	Anxiety	Stress	Sleep Quality
Food insecurity	1										
Academic grading ^a^	−0.080	1									
Perception about overall progress in college	−0.170 ***	0.248 ***	1								
Perception about attendance to classes	−0.185 ***	0.246 ***	0.430 ***	1							
Perception about the ability to pay attention in class	−0.160 ***	0.086	0.401 ***	0.342 ***	1						
Perception about understanding concepts in class	−0.113 *	0.157 **	0.441 ***	0.286 ***	0.552 ***	1					
Perception about academic future	−0.176 ***	0.107 *	0.311 ***	0.160 ***	0.207 ***	0.245 ***	1				
Depression	0.099 *	0.040	−0.243 ***	−0.170 ***	−0.288 ***	−0.278 ***	−0.231 ***	1			
Anxiety	0.130 **	−0.026	−0.210 ***	−0.125 **	−0.193 ***	−0.223 ***	−0.187 ***	0.672 ***	1		
Stress	0.109 *	−0.005	−0.211 ***	−0.138 **	−0.232 ***	−0.187 ***	−0.173 ***	0.690 ***	0.742 ***	1	
Sleep quality	0.135 **	−0.071	−0.182 ***	−0.188 ***	−0.215 ***	−0.179 ***	−0.146 **	0.507 ***	0.459 ***	0.470 ***	1

Spearman’s rho. The correlation analysis was carried out with the four categories of food insecurity (food security, mild FI, moderate FI, and severe FI), the four categories of perception of academic performance (excellent, good, regular, and poor), and the three categories of perception about academic future (successfully complete studies; complete, but with difficulties; not complete the degree). The scores were used for the analysis of depression, anxiety, stress, and sleep quality. * *p* < 0.05 was considered significant. ^a^ First-semester students (*n* = 65) were excluded from the correlation analysis of the academic grading. *** *p* < 0.001, ** *p* < 0.01, and * *p* < 0.05.

**Table 5:** Association of food insecurity (FI), depression, anxiety, stress, and sleep quality with academic performance indicators.

	OR (95% CI) Unadjusted	OR (95% CI) Model I ^a^	OR (95% CI) Model II ^b^	OR (95% CI) Model III ^c^
	Poor/regular perception about overall progress in college, including graduating on time			
Food security	1	1	1	1
Mild FI	2.78 (1.47, 5.28) *	2.64 (1.32, 5.29) *	2.55 (1.28, 5.08) *	2.64 (1.33, 5.24) *
Moderate/severe FI	2.78 (1.46, 5.32) *	2.96 (1.49, 5.88) *	2.85 (1.44, 5.64) *	2.88 (1.46, 5.69) *
Depression	1.10 (1.05, 1.16) *	1.10 (1.04, 1.16) *	---	---
Anxiety	1.08 (1.03, 1.14) *	---	1.05 (0.99, 1.13)	---
Stress	1.07 (1.01, 1.13) *	---	---	1.04 (0.97, 1.11)
Sleep quality	1.10 (1.01, 1.21) *	0.99 (0.88, 1.11)	1.04 (0.93, 1.16)	1.05 (0.94, 1.18)
	Poor/regular perception about attendance to classes			
Food security	1	1	1	1
Mild FI	2.03 (0.76, 5.42)	1.65 (0.55, 4.89)	1.58 (0.53, 4.68)	1.61 (0.55, 4.75)
Moderate/severe FI	3.66 (1.51, 8.84) *	3.14 (1.19, 8.28) *	2.94 (1.12, 7.70) *	2.96 (1.13, 7.75) *
Depression	1.11 (1.04, 1.18) *	1.10 (1.01, 1.19) *	---	---
Anxiety	1.08 (1.004, 1.17) *	---	1.06 (0.96, 1.17)	---
Stress	1.09 (1.01, 1.18) *	---	---	1.07 (0.97, 1.18)
Sleep quality	1.19 (1.05, 1.36) *	1.09 (0.93, 1.28)	1.14 (0.97, 1.33)	1.13 (0.97, 1.32)
	Poor/regular perception about the ability to pay attention in class			
Food security	1	1	1	1
Mild FI	1.75 (1.07, 2.86) *	1.61 (0.94, 2.75)	1.59 (0.94, 2.71)	1.60 (0.94, 2.72)
Moderate/severe FI	1.80 (1.1, 2.96) *	1.59 (0.93, 2.73)	1.57 (0.92, 2.68)	1.58 (0.92, 2.69)
Depression	1.11 (1.07, 1.15) *	1.08 (1.03, 1.13) *	---	---
Anxiety	1.08 (1.03, 1.13) *	---	1.03 (0.98, 1.08)	---
Stress	1.09 (1.04, 1.14) *	---	---	1.05 (0.99, 1.10)
Sleep quality	1.17 (1.08, 1.26) *	1.08 (0.99, 1.18)	1.14 (1.04, 1.24) *	1.12 (1.03, 1.22) *
	Poor/regular perception about the understanding of the concepts in class			
Food security	1	1	1	1
Mild FI	1.40 (0.84, 2.34)	1.34 (0.77, 2.33)	1.27 (0.73, 2.21)	1.35 (0.78, 2.33)
Moderate/severe FI	1.49 (0.89, 2.50)	1.38 (0.79, 2.41)	1.35 (0.78, 2.35)	1.38 (0.79, 2.39)
Depression	1.10 (1.06, 1.15) *	1.08 (1.03, 1.13) *	---	---
Anxiety	1.11 (1.06, 1.16) *	---	1.08 (1.02, 1.14) *	---
Stress	1.08 (1.03, 1.13) *	---	---	1.04 (0.99, 1.09)
Sleep quality	1.12 (1.04, 1.21) *	1.03 (0.94, 1.13)	1.05 (0.96, 1.15)	1.08 (0.99, 1.18)
	Perception about academic future			
Food security	1	1	1	1
Mild FI	1.66 (0.85, 3.24)	1.21 (0.58, 2.52)	1.17 (0.56, 2.42)	1.23 (0.59, 2.53)
Moderate/severe FI	3.29 (1.80, 6.02) *	2.75 (1.43, 5.29) *	2.64 (1.38, 5.05) *	2.63 (1.37, 5.03) *
Depression	1.11 (1.06, 1.16) *	1.09 (1.03, 1.16) *	---	---
Anxiety	1.1 (1.04, 1.16) *	---	1.08 (1.01, 1.15) *	---
Stress	1.1 (1.04, 1.16) *	---	---	1.07 (1.002, 1.14) *
Sleep quality	1.16 (1.05, 1.27) *	1.05 (0.94, 1.18)	1.09 (0.97, 1.21)	1.09 (0.98, 1.22)

OR: Odds Ratio; IC: confidence interval. ^a^ Model I includes the significant variables in the unadjusted analysis, in addition to depression, age, sex, and employment status. ^b^ Model II includes the significant variables in the unadjusted analysis, in addition to anxiety, age, sex, and employment status. ^c^ Model III includes the significant variables in the unadjusted analysis, in addition to stress, age, sex, and employment status. * *p* < 0.05 was considered significant.

## Data Availability

The original contributions presented in the study are included in the article/supplementary material, further inquiries can be directed to the corresponding author.

## Supplementary Materials

The following supporting information can be downloaded at: https://www.mdpi.com/article/10.3390/foods13162508/s1, Table S1: Academic performance according to level of food security/insecurity between women and men; Table S2: Correlations between academic performance and food insecurity, sleep quality, depression, anxiety, and stress symptoms in male university students (*n* = 128); Table S3: Correlations between academic performance and food insecurity, sleep quality, depression, anxiety, and stress symptoms in female university students (*n* = 338); Table S4: Association of food insecurity (FI), depression, anxiety, stress, and sleep quality, with academic performance indicators without first semester students.
