# Supplementary material for: Beyond the Classroom: The Influence of Food Insecurity, Mental Health, and Sleep Quality on University Students’ Academic Performance
Source: Foods. 2024 Aug 11;13(16):2508. doi: 10.3390/foods13162508 (PMC11353649; doi:10.3390/foods13162508)
Supplement: Supplementary file 1 [file foods-13-02508-s001.zip › foods-3100058-supplementary.pdf]

Table S1. Academic performance according to level of food security/insecurity between women and men

	Women							Men						
	Food security		Mild FI		Moderate/severe FI		p	Food security		Mild FI		Moderate/severe FI		p
How would you rate your overall progress in college, including graduating on time? [a]							<0.001*							0.216
Excellent	64	(36.0)	19	(22.6)	18	(23.7)		25	(36.2)	9	(32.1)	7	(22.6)	
Good	102	(57.3)	48	(57.1)	38	(50.0)		35	(50.7)	13	(46.4)	22	(71.0)	
Poor/regular	12	(6.7)	17	(20.2)	20	(26.3)		9	(13.0)	6	(21.4)	2	(6.5)	
How would you rate your attendance (in-person or online) to classes? [a]							0.001*							0.717
Excellent	112	(62.9)	42	(50.0)	28	(36.8)		34	(49.3)	11	(39.3)	14	(45.2)	
Good	61	(34.3)	37	(44.0)	39	(51.3)		31	(44.9)	14	(50.0)	13	(41.9)	
Poor/regular	5	(2.8)	5	(6.0)	9	(11.8)		4	(5.8)	3	(10.7)	4	(12.9)	
How would you rate your ability to pay attention in class? [a]							0.028*							0.048*
Excellent	23	(12.9)	9	(10.7)	3	(3.9)		17	(24.6)	3	(10.7)	3	(9.7)	
Good	114	(64.0)	49	(58.3)	42	(55.3)		37	(53.6)	13	(46.4)	22	(71.0)	
Poor/regular	41	(23.0)	26	(31.0)	31	(40.8)		15	(21.7)	12	(42.9)	6	(19.4)	
How would you rate your understanding of the concepts taught in class? [a]							0.147							0.015*
Excellent	24	(13.5)	12	(14.3)	3	(3.9)		16	(23.2)	3	(10.7)	5	(16.1)	
Good	108	(60.7)	52	(61.9)	47	(61.8)		46	(66.7)	14	(50.0)	21	(67.7)	
Poor/regular	46	(25.8)	20	(23.8)	26	(34.2)		7	(10.1)	11	(39.3)	5	(16.1)	
Regarding your academic future, you consider that you: [a]							<0.001*							0.374
Will successfully complete studies	164	(92.1)	73	(86.9)	56	(73.7)		59	(85.5)	22	(78.6)	23	(74.2)	
Will complete, but with difficulties/ not complete the degree	14	(7.9)	11	(13.1)	20	(26.3)		10	(14.5)	6	(21.4)	8	(25.8)	

Academic grading [b]	95	(93.2, 97)	94.2	(92.6, 96.7)	95	(92.2, 96.9)	0.399	94.8	(90, 97)	94	(92, 97.6)	93	(89, 96)	0.213

[a] The qualitative variables are expressed as frequency (%). Chi-square was performed to evaluate differences between categories.
[b] The quantitative variable is expressed as median (25th 75th percentile). Kruskal Wallis was used to evaluate differences between categories.
*p<0.05 was considered significant.

Table S2. Correlations between academic performance and food insecurity, sleep quality, depression, anxiety, and stress symptoms in male university students (n=128)

	Food insecurity	Academic grading [a]	Perception about overall progress in college	Perception about attendance to classes	Perception about the ability to pay attention in class	Perception about understanding concepts in class	Perception about academic future	Depression	Anxiety	Stress	Sleep quality
Food insecurity	1										
Academic grading [a]	-0.067	1									
Perception about overall progress in college	-0.071	0.468***	1								
Perception about attendance to classes	-0.082	0.410***	0.407***	1							
Perception about the ability to pay attention in class	-0.132	0.108	0.444***	0.384***	1						
Perception about understanding concepts in class	-0.166	0.337***	0.430***	0.327***	0.609***	1					
Perception about academic future	-0.132	-0.049	0.360***	0.123	0.124	0.168	1				
Depression	-0.004	0.159	-0.146	0.037	-0.207*	-0.214*	-0.208*	1			
Anxiety	0.048	0.109	-0.144	-0.065	-0.230**	-0.159	-0.187*	0.590***	1		
Stress	0.064	0.000	-0.261**	-0.149	-0.304***	-0.223*	-0.227*	0.627***	0.738***	1	
Sleep quality	0.127	0.045	-0.150	-0.213*	-0.226*	-0.177	-0.173	0.443***	0.403***	0.437***	1

Spearman's Rho. The correlation analysis was carried out with the four categories of food insecurity (food security, mild FI, moderate FI, and severe FI), the four categories of perception of academic performance (excellent, good, regular, poor), and the three categories of perception about academic future (successfully complete studies; complete, but with difficulties; not complete the degree). The scores were used for the analysis of depression, anxiety, stress, and sleep quality.
* p<0.05 was considered significant
[a] First semester students (n=17) were excluded from the correlation analysis of the academic grading.
***p<0.001, **p<0.01, *p<0.05

Table S3. Correlations between academic performance and food insecurity, sleep quality, depression, anxiety, and stress symptoms in female university students (n=338)

	Food insecurity	Academic grading [a]	Perception about overall progress in college	Perception about attendance to classes	Perception about the ability to pay attention in class	Perception about understanding concepts in class	Perception about academic future	Depression	Anxiety	Stress	Sleep quality
Food insecurity	1										
Academic grading [a]	-0.076	1									
Perception about overall progress in college	-0.209***	0.144*	1								
Perception about attendance to classes	-0.227***	0.162**	0.444***	1							
Perception about ability to pay attention in class	-0.173***	0.076	0.386***	0.337***	1						
Perception about understanding concepts in class	-0.093	0.081	0.449***	0.287***	0.524***	1					
Perception about academic future	-0.197***	0.363***	0.293***	0.171**	0.258***	0.298***	1				
Depression	0.134*	-0.007	-0.278***	-0.252***	-0.313***	-0.292***	-0.248***	1			
Anxiety	0.158**	-0.080	-0.233***	-0.155**	-0.176***	-0.238***	-0.192***	0.699***	1		
Stress	0.128*	-0.019	-0.196***	-0.166**	-0.198***	-0.154**	-0.163**	0.713***	0.752***	1	
Sleep quality	0.139*	-0.128*	-0.192***	-0.184***	-0.206***	-0.171**	-0.143**	0.527***	0.479***	0.482***	1

Spearman's Rho. The correlation analysis was carried out with the four categories of food insecurity (food security, mild FI, moderate FI, and severe FI), the four categories of perception of academic performance (excellent, good, regular, poor), and the three categories of perception about academic future (successfully complete studies; complete, but with difficulties; not complete the degree). The scores were used for the analysis of depression, anxiety, stress, and sleep quality.

* $p<0.05$ was considered significant

[a] First semester students (n=48) were excluded from the correlation analysis of the academic grading.

***p<0.001, **p<0.01, *p<0.05

Table S4. Association between food insecurity (FI), depression, anxiety, stress, and sleep quality, and academic performance indicators without first semester students

	OR (95%CI) Unadjuted	OR (95%CI) Model I [a]	OR (95%CI) Model II [b]	OR (95%CI) Model III [c]
	**Poor/regular perception about overall progress in college, including graduating on time**			
Food security	1	1	1	1
Mild FI	2.84 (1.43, 5.66)*	2.47 (1.17, 5.20)*	2.39 (1.14, 4.99)*	2.46 (1.18, 5.14)*
Moderate/severe FI	2.04 (0.97, 4.31)	2.08 (0.96, 4.53)	1.98 (0.91, 4.30)	1.98 (0.91, 4.31)
Depression	1.06 (1.05, 1.16)*	1.09 (1.02, 1.16)*	---	---
Anxiety	1.10 (1.04, 1.17)*	---	1.06 (0.99, 1.14)	---
Stress	1.09 (1.02, 1.16)*	---	---	1.05 (0.97, 1.13)
Sleep quality	1.14 (1.03, 1.26)*	1.03 (0.90, 1.17)	1.07 (0.95, 1.22)	1.09 (0.96, 1.23)
	**Poor/regular perception about attendance to classes**			
Food security	1	1	1	1
Mild FI	2.71 (0.89, 8.29)	1.98 (0.59, 6.63)	1.87 (0.56, 6.23)	1.91 (0.58, 6.33)
Moderate/severe FI	4.91 (1.76, 13.73)*	3.55 (1.19, 10.59)*	3.23 (1.08, 9.62)*	3.24 (1.09, 9.67)*
Depression	1.11 (1.03, 1.19)*	1.10 (1.01, 1.20)*	---	---
Anxiety	1.11 (1.02, 1.20)*	---	1.08 (0.97, 1.20)	---
Stress	1.11 (1.02, 1.22)*	---	---	1.09 (0.98, 1.22)
Sleep quality	1.19 (1.03, 1.38)*	1.08 (0.90, 1.29)	1.12 (0.93, 1.33)	1.12 (0.94, 1.33)
	**Poor/regular perception about the ability to pay attention in class**			
Food security	1	1	1	1
Mild FI	1.62 (0.96, 2.76)	1.43 (0.80, 2.56)	1.45 (0.81, 2.57)	1.42 (0.80, 2.53)
Moderate/severe FI	1.51 (0.88, 2.62)	1.25 (0.68, 2.28)	1.23 (0.68, 2.23)	1.21 (0.66, 2.20)
Depression	1.11 (1.06, 1.15)*	1.07 (1.02, 1.13)*	---	---
Anxiety	1.08 (1.03, 1.13)*	---	1.02 (0.97,1.08)	---
Stress	1.10 (1.05, 1.15)*	---	---	1.06 (0.99, 1.12)
Sleep quality	1.17 (1.08, 1.27)*	1.09 (0.99, 1.21)	1.15 (1.04, 1.27)*	1.12 (1.02, 1.24)*
	**Poor/regular perception about the understanding of the concepts in class**			
Food security	1	1	1	1
Mild FI	1.39 (0.79, 2.44)	1.34 (0.73, 2.46)	1.26 (0.68, 2.33)	1.35 (0.74, 2.48)
Moderate/severe FI	1.16 (0.64, 2.11)	1.05 (0.55, 2.02)	1.01 (0.53, 1.94)	1.04 (0.55, 1.99)
Depression	1.09 (1.04, 1.14)*	1.07 (1.01, 1.13)*	---	---
Anxiety	1.11 (1.06, 1.17)*	---	1.09 (1.03, 1.16)*	---
Stress	1.07 (1.02, 1.12)*	---	---	1.04 (0.98, 1.10)
Sleep quality	1.11 (1.02, 1.21)*	1.02 (0.92, 1.14)	1.02 (0.92, 1.13)	1.07 (0.96, 1.18)
	**Perception about academic future**			
Food security	1	1	1	1
Mild FI	1.36 (0.65, 2.82)	0.92 (0.40, 2.10)	0.90 (0.40, 2.06)	0.93 (0.41, 2.12)

Moderate/severe FI	2.71 (1.40, 5.23)*	2.16 (1.05, 4.47)*	2.02 (0.98, 4.17)*	1.99 (0.96, 4.12)*
Depression	1.12 (1.06, 1.17)*	1.10 (1.03, 1.18)*	---	---
Anxiety	1.11 (1.05, 1.18)*	---	1.08 (1.01, 1.16)*	---
Stress	1.13 (1.06, 1.20)*	---	---	1.1 (1.02, 1.18)*
Sleep quality	1.18 (1.07, 1.31)*	1.08 (0.95, 1.22)*	1.12 (0.98, 1.26)	1.11 (0.98, 1.26)*

OR: Odds Ratio; IC: confidence interval.

[a] The model I includes the significant variables in the unadjusted analysis, in addition to depression, age, sex and employment status.

[b] The model II includes the significant variables in the unadjusted analysis, in addition to anxiety, age, sex and employment status.

[c] The model III includes the significant variables in the unadjusted analysis, in addition to stress, age, sex and employment status

* $p<0.05$ was considered significant
